# The Impact of Molecular Symmetry and Rigidity on the Selective Analysis of VOCs by Mid-IR Laser Spectroscopy

**DOI:** 10.1021/acs.analchem.5c02084

**Published:** 2025-06-14

**Authors:** Miloš Selaković, Renato Zenobi, Lukas Emmenegger, Béla Tuzson

**Affiliations:** † 28501 Laboratory for Air Pollution/Environmental Technology, Empa, Überlandstrasse 129, 8600 Dübendorf, Switzerland; ‡ 27219 Department of Chemistry and Applied Biosciences, ETH Zurich, Vladimir-Prelog-Weg 3, 8093 Zurich, Switzerland

## Abstract

This study highlights the potential of mid-infrared laser absorption spectroscopy (LAS) for analyzing volatile organic compounds (VOCs) in the gas phase. Using a Vernier-type quantum cascade laser (QCL) in the 9–10 μm spectral region, over 40 different VOCs were systematically investigated with a spectral resolution better than 0.005 cm^–1^. It was found that many VOCs, even with more than six heavy atoms, can exhibit significant spectral features at ambient temperature and pressures above 10 mbar, i.e., at conditions well suited for routine analysis. Our observations suggest that in addition to molecular weight and number of atoms, molecular rigidity and symmetry have a decisive impact on the spectral feature content. The high spectral feature content observed in rigid molecules is related to their small number of conformers, while higher molecular symmetry results in fewer nondegenerate vibrational modes, principal rotational axes with different moments of inertia, and a reduced number of nonidentical conformers. Overall, this leads to distinct spectral features that enable the selective and sensitive detection of large VOCs by high-resolution LAS.

## Graphical abstract

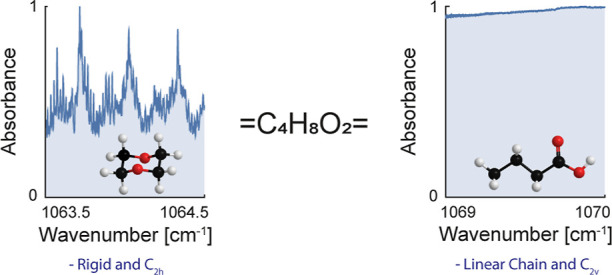

## Introduction

Analysis of trace amounts of volatile organic compounds (VOCs) in the gas phase has gained substantial importance in a large variety of applications, covering areas such as industrial quality control,^1,2^ environmental monitoring,^3,4^ and medical diagnostics.^5,6^ The molecular weight of the targeted VOCs can substantially differ, from relatively small VOCs with one to four carbon atoms (C1–C4), for example, when detecting spoiled food,^2^ up to compounds with molecular weights over 200 Da in the case of polyhalogenated hydrocarbons, relevant for air quality monitoring.^3^ Currently, the most widely deployed analytical techniques for VOC quantification are gas chromatography and mass spectrometry. While these techniques provide low detection limits for a broad range of VOCs,^7^ they are often limited with respect to size, cost, or speed. Thus, alternative approaches are needed, especially for routine analysis, which can deliver a rapid and precise response in compact, easy-to-use, and cost-effective instrumentation.

Spectroscopic detection of VOCs in the mid-infrared (mid-IR) range stands out as a viable alternative to meet all these requirements. This approach exploits the unique fingerprint absorption of organic compounds originating from the most intense vibrational bands in the spectral range of 2.5–25 μm. To date, the largest number of spectroscopic parameters has been retrieved from Fourier transform spectroscopy (FTS). This technique enables access to highly resolved (≈0.001 cm^–1^) ro-vibrational bands of breath-related VOCs such as methanol, ethanol, isoprene, and acetylene, as well as VOCs with astrochemical relevance, such as vinyl bromide (CH_2_CHBr), adamantane (C_10_H_16_), and diamantane (C_14_H_20_).^8^ When combined with low-temperature approaches such as supersonic expansion jet^9^ or buffer-gas-cooled^10^ techniques, even highly complex molecules, e.g., fullerenes with a molecular mass of 720 Da, reveal fine spectroscopic features.^11^ While these methods are highly valuable, their application is largely constrained to fundamental research due to the complexity of the involved equipment.

To provide fast and compact instrumentation for routine analysis, such as medical diagnostics or industrial quality control, a more cost-effective approach is needed that is applicable to easily accessible measurement conditions. Bench-top Fourier-transform infrared (FTIR) spectrometers are viable alternatives, though with much lower spectral resolution, typically of about 0.1 cm^–1^, that is not sufficient to fully explore the spectral features of VOCs in the gas phase.

Dual-comb spectroscopy (DCS) is emerging as a new spectroscopic tool that exploits the frequency resolution, frequency accuracy, broad bandwidth, and brightness of frequency combs,^12^ overcoming sensitivity limitations of conventional FTIR spectrometers. First demonstrations of its capabilities for studying VOC molecules that play an important role in medical breath research are very promising.^13^ However, the DCS technique remains challenging in the mid-infrared region due to difficulties in sources, requiring complex and expensive setup.^14^ Furthermore, although powerful in many respects of spectroscopic performance, the signal-to-noise ratio and detection limits of DCS systems are still inferior compared to direct absorption laser spectroscopy (often called LAS) based on single-mode and continuous-wave light sources, e.g., quantum-cascade lasers (QCLs).^15^

The performance of LAS has been demonstrated widely in both research-grade and commercial systems, albeit mainly for greenhouse gases (GHGs) and air pollutants, including small VOCs such as formaldehyde.^16^ In a recent work, Brechbühler et al.^17^ showed that selective VOC analysis at an amount fraction level of a parts-per-billion (ppb) can be performed using LAS whenever characteristic spectral features are available. This demonstration, however, was limited to methanol, ethanol, and acetaldehyde, i.e., relatively small VOCs. Arguably, the analysis of larger and more complex organic molecules by LAS remains challenging and is feasible only if they exhibit sufficient spectral information.

For practical reasons, here we adopt the term *large*, referring to a compound that has a molecular weight above 40 Da^18^ or contains four or more heavy (non-hydrogen) atoms.^19^ It is widely accepted that the ro-vibrational absorption spectra of these compounds at trivial measurement conditions, such as room temperature and rough vacuum, lack specific features which would be needed for selective detection and quantitative measurements.^19–22^ This is usually attributed to the increasing number of vibrational modes that scales with the number of atoms in the molecule, large moment of inertia, and overtone bands resulting in spectral congestion.^18,23^

In this paper, we challenge this paradigm by demonstrating that even VOCs with more than six heavy atoms or with a molecular weight over 100 Da can exhibit significant spectral features in their mid-IR ro-vibrational absorption spectra under easily accessible experimental conditions so that they can be selectively quantified with narrow-band laser spectroscopy. Furthermore, we present a qualitative exploration of parameters that, besides the molecular weight, can explain the presence and characteristics of spectral features of VOCs in the gas phase.

## Methods

### QCL Spectroscopy

The Vernier QCL-based spectrometer as well as the laser-driving scheme were described in detail previously.^17^ In brief, a prototype extended-tuning QCL (QC-XT, Alpes Lasers) was coupled to a 76 m multipass cell (AMAC-76, Aerodyne Research, Inc.), and the transmitted beam was sensed by a thermoelectrically cooled IR detector (PVMI-4TE-8, Vigo Photonics S.A.). The Vernier laser can be operated within six spectral windows distributed between 1063 cm^–1^ and 1102 cm^–1^. In each window, it behaves as a free-running distributed feedback (DFB)-type laser with a spectral resolution better than 0.005 cm^–1^. A complete spectrum covering all individual windows was recorded every 360 ms by rapid switching between and frequency tuning within the six windows with a pulse repetition rate of 3.33 kHz. The detector signal was coaveraged on board by an FPGA (Alpha250, Koheron) and transferred to a PC at a 1 Hz data rate for further spectral processing.

### Gas-Delivery System

The multipass cell (MPC) was connected upstream to a multiport selector (EUTB-2SD6MWE, VICI AG International) to switch between a VOC gas standard, CO_2_, and N_2_. The CO_2_ (grade 4.5, PanGas) served for the absolute-frequency calibration, while N_2_ (grade 5.0, PanGas) was used for recording background spectra. Mass-flow controllers (GSC series, Vögtlin Instruments GmbH) were used to establish constant CO_2_ and N_2_ flow rates of 80 and 200 mL/min, respectively. Downstream, the MPC was connected to a pressure controller (PC series, Alicat) and a vacuum pump (N920 KT, KNF). The pressure and temperature in the MPC were measured with an absolute pressure transducer (Baratron 722B, MKS Instruments, Inc.) and a 10 kΩ thermistor, respectively.

For gas-phase VOC sample generation, a commercial evaporation system (HovaCAL 424-VOC4, IAS GmbH) was employed. All chemicals used in this study had a purity of better than 95%. Whenever low-volatility, solid, or corrosive chemicals were investigated, an alternative sampling scheme was employed. If these analytes were sufficiently soluble in water, then an aqueous solution was used for dosing into the calibration unit. Otherwise, compound vapors at room temperature or slightly elevated temperature were diluted in nitrogen and introduced to the spectrometer.

### Spectral Analysis

The raw spectrum, *I*(*j*), was normalized by the background signal, *I*_0_(*j*), i.e., by the spectrum measured when the MPC was purged with N_2_, and then the transmission spectra, *T*(*j*), were calculated. The spectral-index scale was converted into a relative wavenumber scale, ν̃(*j*), by using the transmission spectrum of a 2 inch-long Ge etalon and an Akima spline as a mapping function.^24^ To establish the absolute wavenumber scale, ν̅(*j*), the transmission spectrum of CO_2_, T(ν̃(j)) , was used by anchoring the spectrum to line positions taken from the HITRAN database.^25^ The absorbance spectra for each investigated VOC were obtained from the transmittance: $A\left(\mathrm{\nu ̅}\left(j\right)\right)=-\log_{10}\left[T\left(\mathrm{\nu ̅}\left(j\right)\right)\right]$ , and then the relative absorbance scale was defined as $Ã\left(\mathrm{\nu ̅}\left(j\right)\right)=A\left(\mathrm{\nu ̅}\left(j\right)\right)/\max_{k}A\left(\mathrm{\nu ̅}\left(k\right)\right)$ . The relative absorbance scale was used to avoid uncertainties related to the purity of the reference materials. The typical noise floor of spectra recorded at a time resolution of 360 ms was about 1 × 10^–3^. Thus, the standard uncertainty of the relative absorbance for all compounds investigated in this study ranged, depending on the scaling factor, between 0.0004 and 0.01 (see, e.g., the specific case of butyric acid illustrated in Figure S1). The signal-to-noise ratio can further be improved by increasing the integration time over 60 s, as demonstrated previously by Brechbühler et al.^17^ The data processing was done with custom-built MATLAB scripts.

### Conformational Study of *N*-Methylformamide (NMF)

To enrich the content of the *cis*-NMF, the photochemical approach from ref 26 was adopted. The compound was irradiated with UV light from a 3 W low-pressure mercury-vapor lamp, and the sample headspace was transferred to the MPC. The cell was closed, and the IR spectra were recorded over ∼20 min. To decompose the time series into concentration profiles and spectra of *cis* and *trans* conformers, multivariate curve resolution-alternating least-squares (MCR-ALS) were used.^27^

## Results and Discussion

The high-resolution absorption spectra of over 40 VOCs at an amount fraction of about 200 ppm were recorded at easily accessible conditions, i.e., at room temperature and at a gas pressure of 10 mbar in the spectral range between 1063 cm^–1^ and 1102 cm^–1^. While the exact values of the individual spectral windows were mainly determined by the laser source, the advantage of working in the 9–10 μm range is that the absorption bands in the longer wavelength region have a well‑resolved ro-vibrational structure compared to the C–H and O–H stretch spectral region at around 3 μm, where the density of vibrational states forms a vibrational quasi-continuum.^28,29^

As shown in the example of ethanol in Figure 1a, the high-resolution absorption spectrum of a VOC can be divided into two sections: (i) bulk absorption and (ii) spectral fingerprint with resolved structural details. The latter is essential for quantitative analysis, since it contains the compound-specific spectral information. In order to quantify this aspect, we propose a basic metric, the spectral feature content, that is defined as the ratio of the integrated area of distinct absorption features over the total area of the spectrum. The challenge of determining this metric is, however, to find a robust method that can identify the bulk component of a spectrum in an objective and reproducible manner. Similarly to baseline fitting methods, we seek an approach that allows for a reliable separation of the bulk from the feature-rich absorption without the need of making any assumptions about spectral characteristics. Therefore, we developed an algorithm that relies on the following hypotheses: (i) for a sufficiently narrow spectral range, e.g., <2 cm^–1^ for conventional DFB lasers, the envelope of the bulk absorption of a spectrum can be approximated with a low-order polynomial, and (ii) there is a unique polynomial of a fixed order that does not exceed the absorption spectrum at any point and the integral of which is maximal within the given spectral range. In particular, we used a custom-developed algorithm to separate the spectral features based on a third-order polynomial.

**Figure 1:**
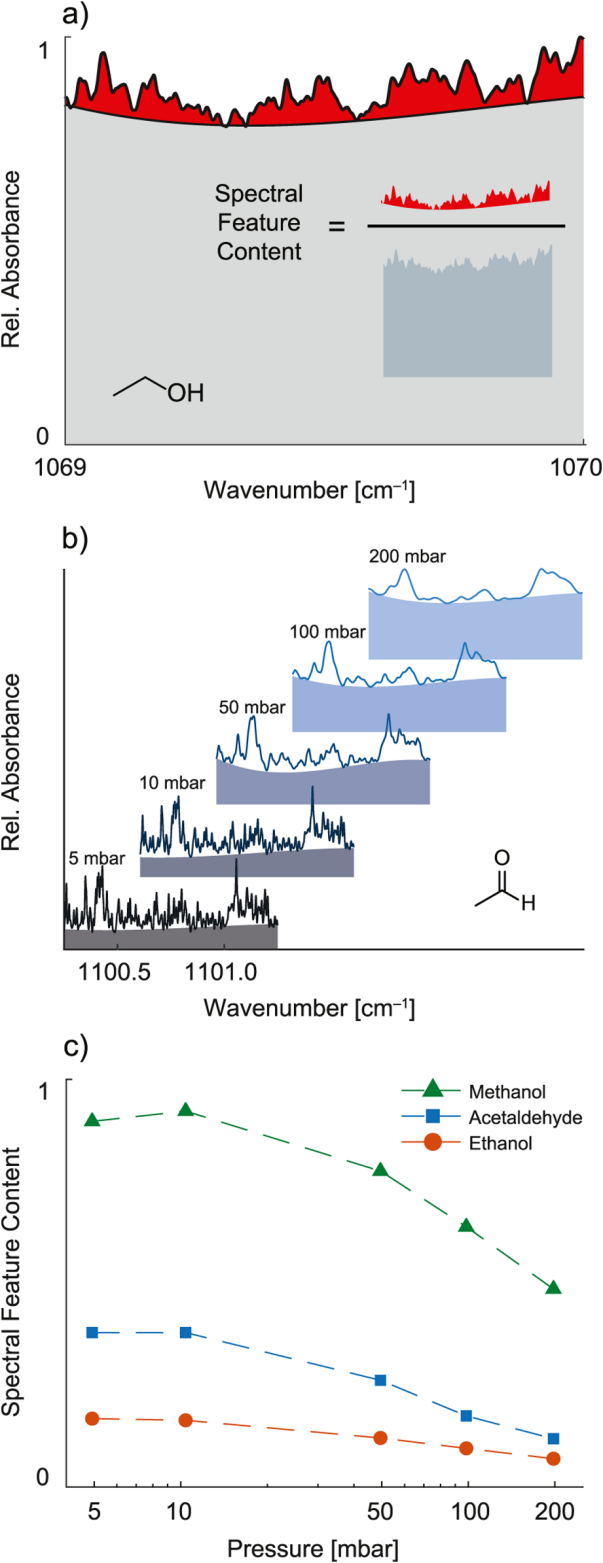
Spectral feature content of methanol, ethanol, and acetaldehyde. (a) The IR spectrum of ethanol can be divided into a high-resolution fingerprint (red) and a bulk absorption (gray) section. As indicated in the inset, the metric of spectral feature content is defined as the ratio of the area of the fingerprint and the area under the whole spectrum. (b) IR spectra of acetaldehyde recorded at different pressures along with the estimated bulk absorption indicated by the shaded areas. (c) Spectral feature content of methanol, ethanol, and acetaldehyde as a function of gas pressure.

As the absorption pattern for any given compound is influenced by the pressure due to collisional broadening, it is favorable to perform the analysis at reduced gas pressure. To establish the optimal conditions to study molecular factors that influence the feature content, spectra of representative compounds (methanol, ethanol, and acetaldehyde) were recorded at different pressures. As indicated in Figure 1 (panels b and c), the spectral feature content of the VOCs is increasing as the gas pressure drops and then levels off when the Doppler broadening starts dominating the collisional broadening. Further decrease of the pressure only reduces the absorption strength without a substantial change in the spectral feature content. Consequently, we selected 10 mbar as the optimal pressure for this study.

As indicated in Figure 2a, the spectral feature content rapidly decreases and eventually almost disappears in the case of a homologous series of linear-chain carboxylic acids. The same trend is observed for straight-chain alcohols and aldehydes, as depicted in Figure 2b. This behavior is expected according to the literature, and, as mentioned before, it is attributed to the number of vibrational modes^18^ that linearly increase in homologous series.

**Figure 2:**
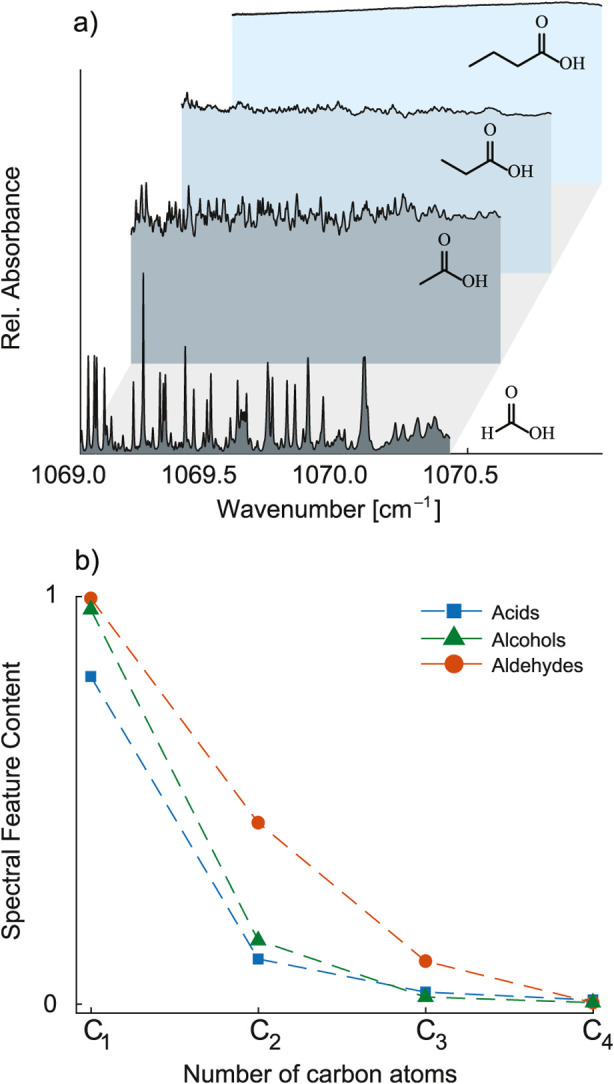
Spectral feature content of a homologous series of compounds. (a) IR spectra of a homologous series of carboxylic acids. (b) Spectral feature content as a function of number of carbon atoms for three homologous series of compounds (alcohols, aldehydes, and carboxylic acids). The spectrum of formaldehyde in the range of 1768–1770 cm^–1^ was simulated using line-by-line parameters from the HITRAN database.^25^

However, the number of vibrational modes and molecular weight of a compound cannot entirely explain the absence of spectral feature content. This is illustrated by 1,3- and 1,4-dioxane (Figure 3), which are isomers of butyric acid (Figure 2a). The dioxanes have the same number of fundamental vibrational modes and molecular weight. However, although butyric acid has nearly no spectral fine structure, the dioxanes exhibit unique structure-rich absorption spectra. Similarly, spectral screening of over 40 different VOCs revealed that many other large VOCs with over five non-hydrogen atoms show very distinct, well-resolved spectral features (some examples are given in Figure 3). Note that for each compound, the spectral window with the most prominent absorption features is displayed. Based on the mid-IR spectra of this study and a general understanding of the molecular characteristics that determine mid-IR absorption, we propose the following two qualitative rules: (i) rigid chemical structures lead to higher spectral feature content, and (ii) molecules with higher symmetry tend to have specific absorption patterns.

**Figure 3:**
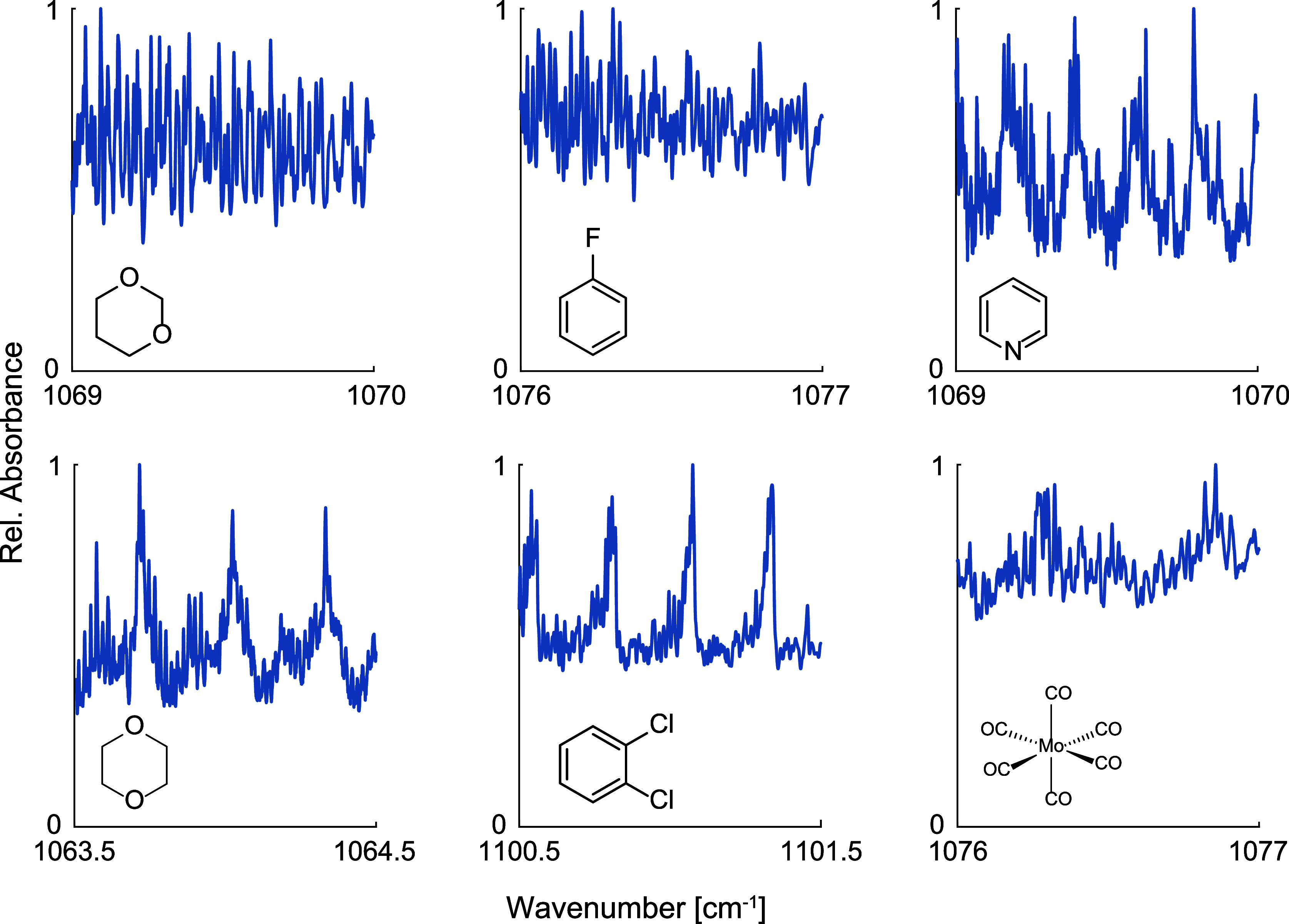
High-resolution IR spectra of several compounds (1,3-dioxane, 1,4-dioxane, fluorobenzene, 1,2-dichlorobenzene, pyridine, and hexacarbonylmolybdenum(0)) with 6 or more non-hydrogen atoms.

To investigate the effect of molecular rigidity on the spectral feature content, the IR spectra of several compounds with similar molecular weights but different chemical structures were measured. Figure 4a depicts such an example. The five compounds were selected to have the same skeleton (C–C–O–C–C) and comparable molecular weights ranging from 68 to 74 Da, yet with significantly different levels of rigidity. Namely, diethyl ether has a very flexible chemical structure with a relatively low rotational barrier for each C–C or C–O bond. This implies that this compound has 81 possible conformers, among which 4 are spectroscopically distinguishable, and that it can easily exchange between them. In comparison, ethyl vinyl ether has a more constrained chemical structure since the rotation around the double bond is not permitted and the rotation around C­(sp^2^)–O is hindered.^30^ Therefore, this molecule has fewer available conformers, i.e., 18, among which 4 are spectroscopically distinguishable. Tetrahydrofuran has even fewer conformers (4, among which two are nonidentical) since the full rotation around the C–C or C–O bond is not possible any longer, and the conformational changes are limited to ring puckering.^31^ An even more confined structure is present in 2,5-dihydrofuran, where ring puckering is limited to the oxygen atom flipping in and out of the C_4_ plane, leading to only two possible identical conformers.^32^ Finally, furan has the most rigid structure, with only one available conformer. As shown in Figure 4a, the spectral feature content is correlated with molecular rigidity, i.e., the more rigid the molecule, the higher the spectral feature content. Their relation is given in a log–log plot (Figure 4b), where, as a simplified measure of the rigidity, the number of conformers is used. To have a better quantitative representation, it would be essential to take into account not only the number of conformers but also their distribution, i.e., the energy of individual conformers,^33^ which is often extremely time-consuming to compute.

**Figure 4:**
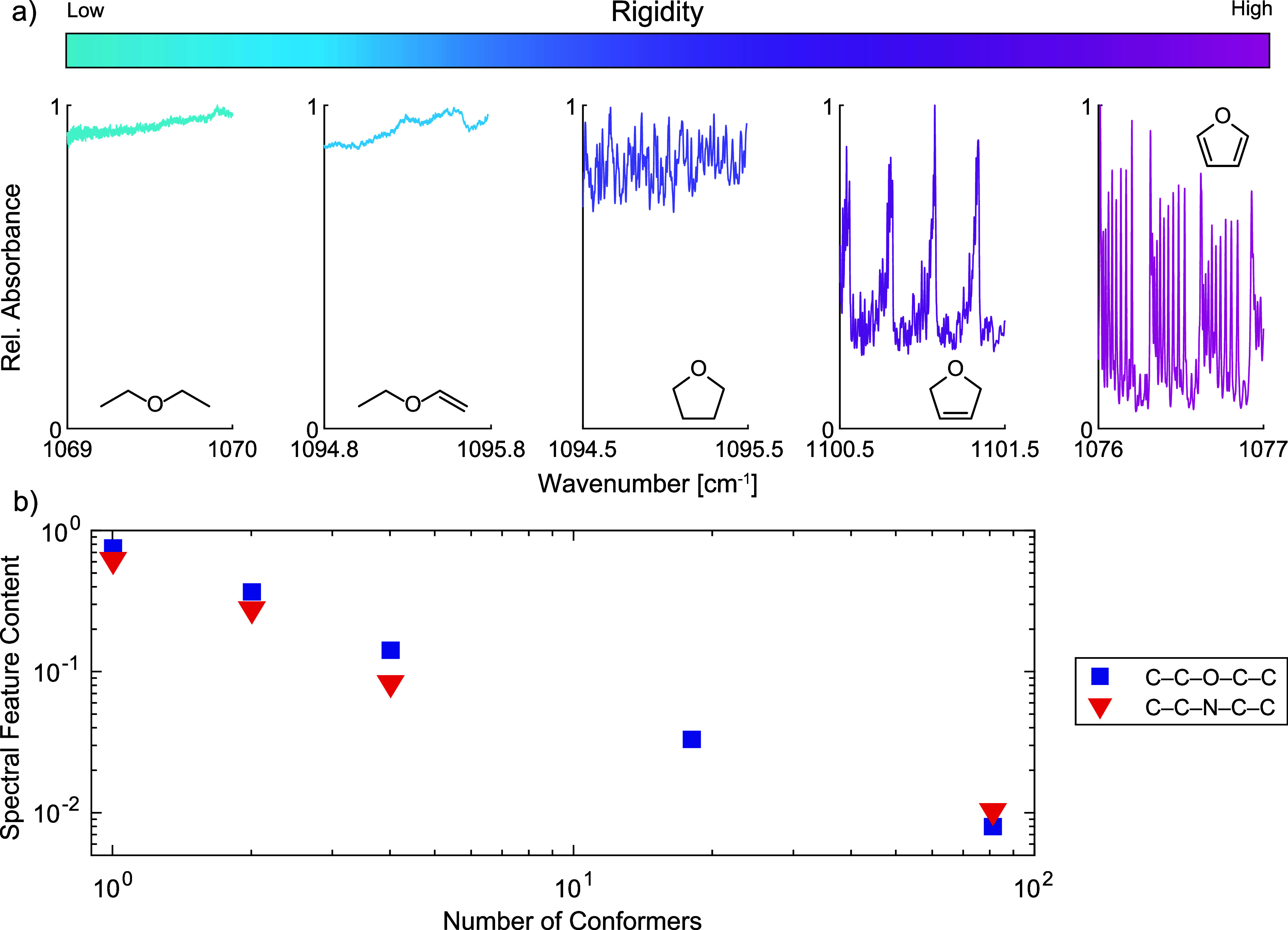
Effect of structural rigidity on spectral feature content. (a) IR spectra (left to right) of diethyl ether, ethyl vinyl ether, tetrahydrofuran, 2,5‑dihydrofuran, and furan. (b) Log–log plot of spectral feature content of compounds with the same skeleton as a function of the number of their conformers. The blue series refers to compounds given in panel (a). The red series refers to diethylamine, pyrrolidine, 2,5-dihydro-1*H*-pyrrole, and pyrrole.

The observation that the spectral feature content is affected by the number of conformers, even in such a narrow spectral domain, indicates that laser spectroscopy can be used for conformational analysis. To demonstrate this, we measured the conformers of NMF. This compound exists in two forms—a more stable, *trans*, and higher-energy, *cis* form, as shown in Figure 5a. At room temperature, the transition rate between the two conformers is relatively slow.^34^ Also, the equilibrium is greatly shifted to favor the *trans* form. Therefore, the absorption spectrum of well-equilibrated NMF vapors mainly contains the contribution of the *trans* form (Figure 5b). Accordingly, the absorption spectrum of a sample that has been photochemically enriched in the *cis* conformer (see Figure 5c) is a firm indication that the two forms have clearly distinguishable ro-vibrational features. After a re-equilibration time of about 20 min, the recorded absorption spectrum (Figure 5d) matches the initial spectrum (Figure 5b). The time series of re-equilibration (Figure 5e) can be decomposed into the spectra of *cis* and *trans* conformers (given in Figure 5f) by applying MCR-ALS. The concentration profile of the *cis* conformer was used to validate the kinetic model of reversible reaction (Figure 5g). According to the Eyring equation^35^ and the reaction rate constant, the activation energy of the process was estimated to be ∼20.9 kcal/mol. This is in good agreement with a predicted value of ∼19.9 kcal/mol.^34^

**Figure 5:**
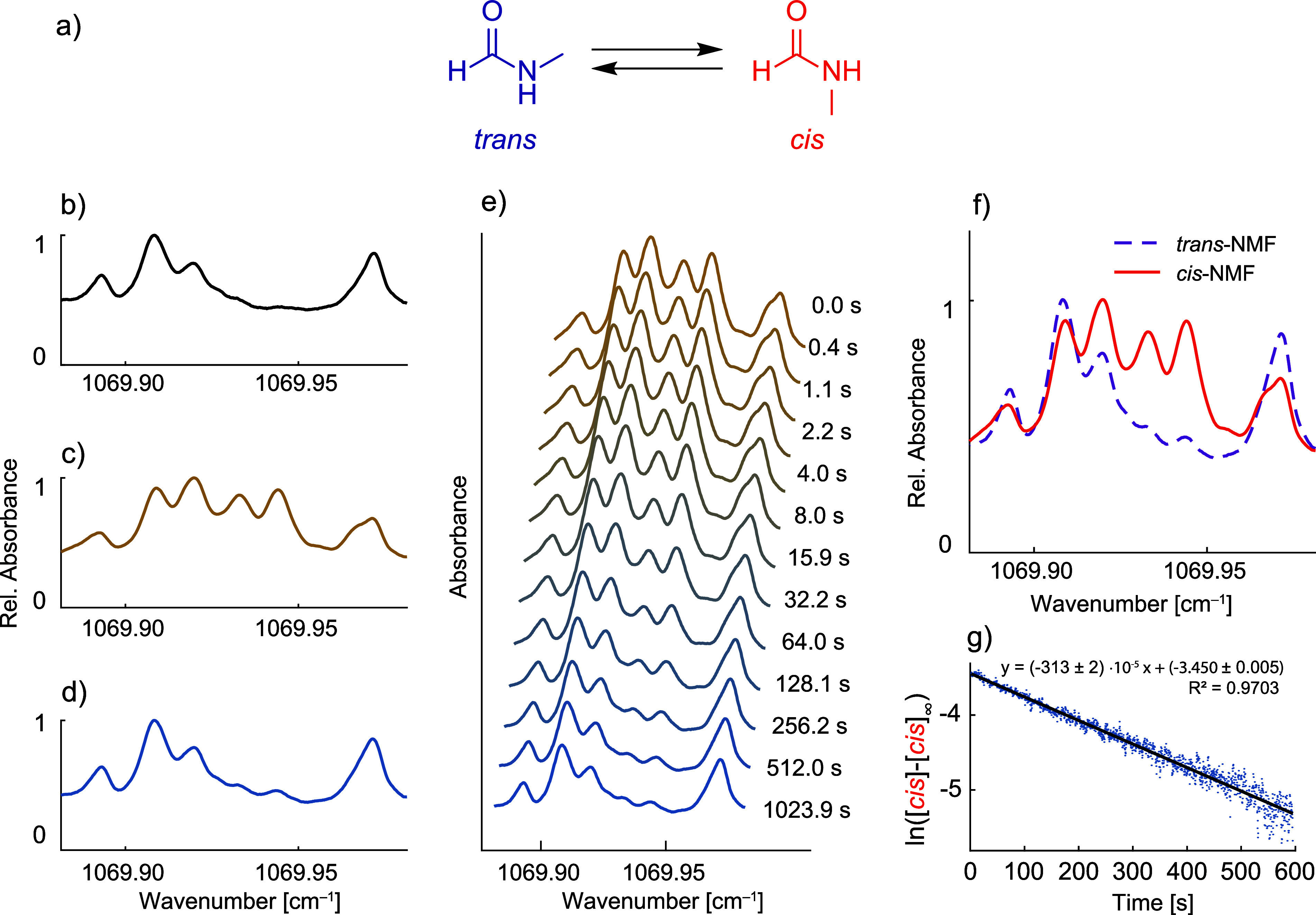
Monitoring of *N*-methylformamide (NMF) conformational transition with laser spectroscopy. (a) Reaction of the conformational exchange of NMF. (b) IR spectrum of NMF vapors at room temperature and 10 mbar. (c) Same as (b) but with the *cis* conformer photochemically enriched (see the Methods section). (d) IR spectrum of the NMF sample originally enriched with the *cis* conformer after ∼20 min. (e) Time series (∼17 min) of NMF absorption spectra, originally enriched with its *cis* conformer. By applying multivariate curve resolution-alternating least-squares (MCR-ALS), the time series is decomposed into spectra of *cis* (orange) and *trans* (purple) conformers (panel f) as well as their concentration profiles. (g) Kinetic model of reversible reaction fitted to the concentration profile of the *cis* conformer.

To investigate the effect of molecular symmetry on the spectral feature content, the IR spectra of several representative compounds with similar molecular weight and rigidity that belong to different symmetry groups have been measured. One such example is displayed in Figure 6a. The three compounds studied, 1,2-, 1,3-, and 1,4-difluorobenzene, are positional isomers belonging to C_2v_, C_2v_, and D_2h_ point groups, respectively. The *para* isomer is more symmetric, having three rotational *C*_2_ axis, three mirror planes, and an inversion point, while *ortho* and *meta* isomers have one C_2_ rotational axis and two mirror planes. This difference in molecular symmetry is also reflected in their ro-vibrational spectra, where the *para* isomer has a significantly higher spectral feature content than the other two isomers. There are several ways in which molecular symmetry can influence the spectral feature content:

1. Through its impact on the number of nondegenerate vibrational modes. This is presumably the main cause for the difference in the fine structure of the positional isomers of the substituted aromatic compounds.
2. By affecting the number of the principal rotational axes with different moments of inertia. Molecules that belong to point groups C_∞v_, D_∞h_, T_d_, or O_h_ have a unique moment of inertia about all principal axes, while molecules that belong to, for example, point groups C_1_, C_s_, C_i_ or C_n_ have three principal rotational axes with three different moments of inertia. As a consequence, the first class of highly symmetric molecules possesses a lower density of ro-vibrational lines and higher spectral feature content compared to the second class of compounds. Figure 6b shows such an example.
3. By affecting the number of spectroscopically distinguishable conformers. In Figure 6c, the IR spectra of two isomers of methyl-1,3-dioxolane are shown, where the 2-methyl isomer has a noticeably more pronounced fine structure than the 4-methyl isomer. While both compounds have four conformers, all four are nonidentical in the case of the 4-methyl isomer, whereas 2-methyl-1,3-dioxolane has three spectroscopically distinguishable conformers due to the existence of a mirror plane.

**Figure 6:**
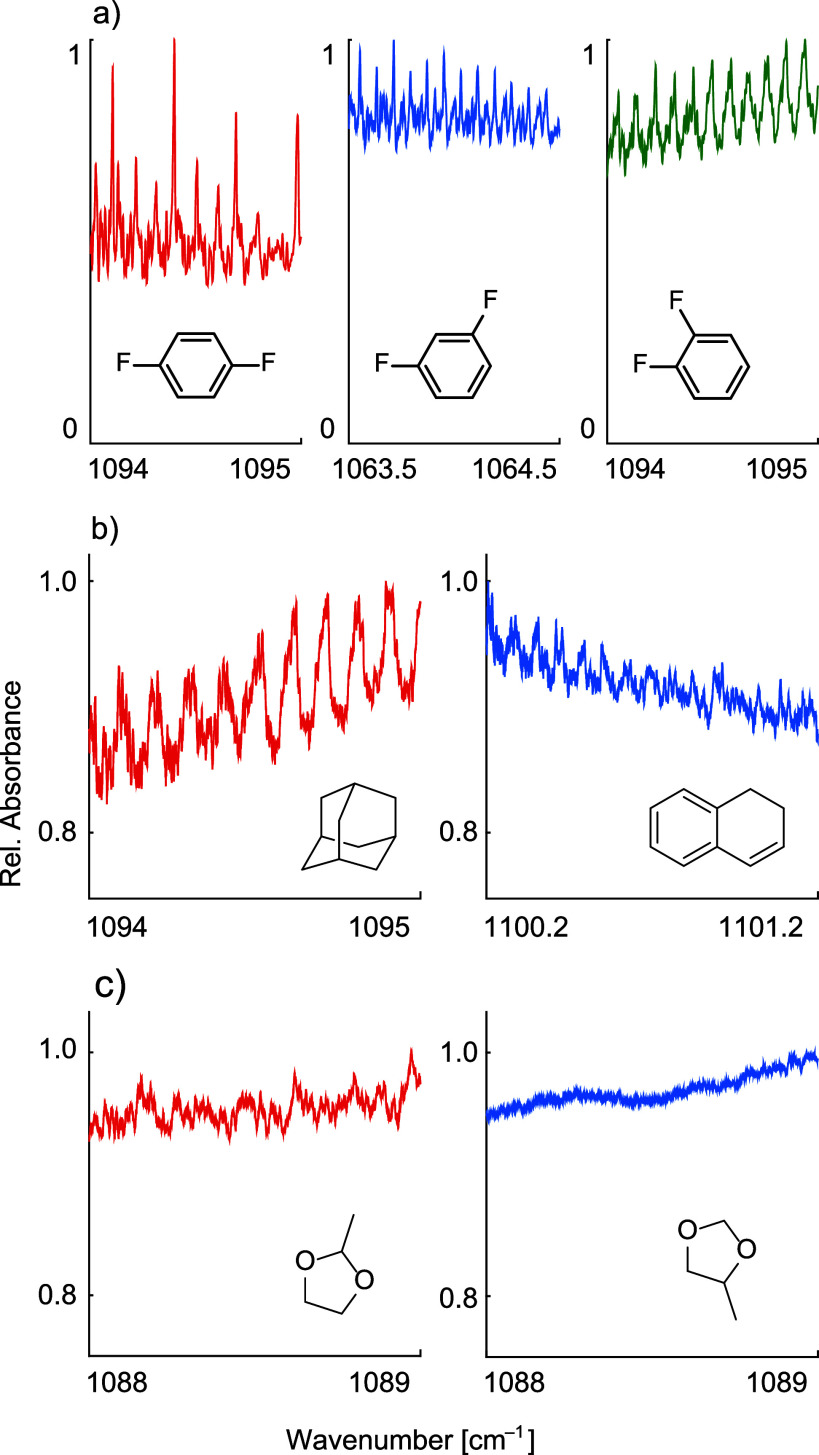
Effect of molecular symmetry on spectral feature content. (a) The high-resolution IR spectra of *p*- (red), *m*- (blue), and *o*-difluorobenzene (green). The *para* isomer, as a more symmetric molecule, has a significantly higher spectral feature content than the other two isomers, which is most likely related to the number of nondegenerate vibrational modes. (b) Adamantane (red), with a unique moment of inertia about all principal rotational axes, has much higher spectral feature content in its IR spectrum than 1,2-dihydronaphthalene (blue) with three principal axes and moments of inertia. (c) The high-resolution IR spectra of 2-methyl (red) and 4-methyl-1,3-dioxolane (blue). The difference in the number of spectroscopically distinguishable conformers is reflected in different spectral feature content of the two isomers.

The 9–10 μm region is rich in distinguishable spectral signatures belonging to fundamental bands of several organic molecules with relevance to applied spectroscopy. Our future work will exploit the QC-XT-based laser spectrometer for analysis of large VOCs in complex matrices such as human breath. Our aim is to develop and apply a reliable spectroscopic method to in situ detect and assess the relevance of potential biomarkers for asthma in young children’s exhaled breath.

## Conclusions

A systematic spectral survey of a multitude of VOCs revealed that many large VOCs with four or more heavy atoms display distinct spectral features in their mid-IR spectra, which can be effectively resolved by LAS. Furthermore, we showed that the feature content in a VOC spectrum is largely controlled by the structural rigidity and molecular symmetry of the compounds. This challenges the widely accepted view according to which the molecular weight or number of atoms are the dominating factors. Consequently, our findings substantially broaden the scope of potential analytes in LAS, paving the way for diverse applications in medical diagnostics, environmental monitoring, and industrial quality control. In particular, given the impact of structural rigidity, many aromatic compounds are suitable targets for analysis by mid-IR spectroscopy. This is especially beneficial for analysis of monoaromatic hydrocarbons (such as benzene, toluene, ethylbenzene, and xylene) in the oil and petroleum industry.

Furthermore, we demonstrate that our approach can be used for the selective measurement of different conformers of a VOC. Because the measurements are performed in the gas phase at reduced pressure, conformational studies done with LAS may be used for constraining computational methods and become a valuable tool for mechanistic studies in the gas phase.

## Supplementary Material



## Notes

Absorbance spectra (PDF)
