# Supplementary material for: The Impact of Molecular Symmetry and Rigidity on the Selective Analysis of VOCs by Mid-IR Laser Spectroscopy
Source: Anal Chem. 2025 Jun 14;97(25):13586–93. doi: 10.1021/acs.analchem.5c02084 (PMC12224160; doi:10.1021/acs.analchem.5c02084)
Supplement: Supplementary file 1 [file ac5c02084_si_001.pdf]

Supporting Information
The impact of molecular symmetry and rigidity on the selective analysis of VOCs by mid-IR laser spectroscopy

Miloš Selaković,[†,‡] Renato Zenobi,[‡] Lukas Emmenegger,[†] and Béla Tuzson[*,†]

†*Laboratory for Air Pollution / Environmental Technology, Empa, Überlandstrasse 129, 8600 Dübendorf, Switzerland*

‡*Department of Chemistry and Applied Biosciences, ETH Zurich, Vladimir-Prelog-Weg 3, 8093 Zurich, Switzerland*

E-mail: bela.tuzson@empa.ch

Phone: +41 58 765 4642

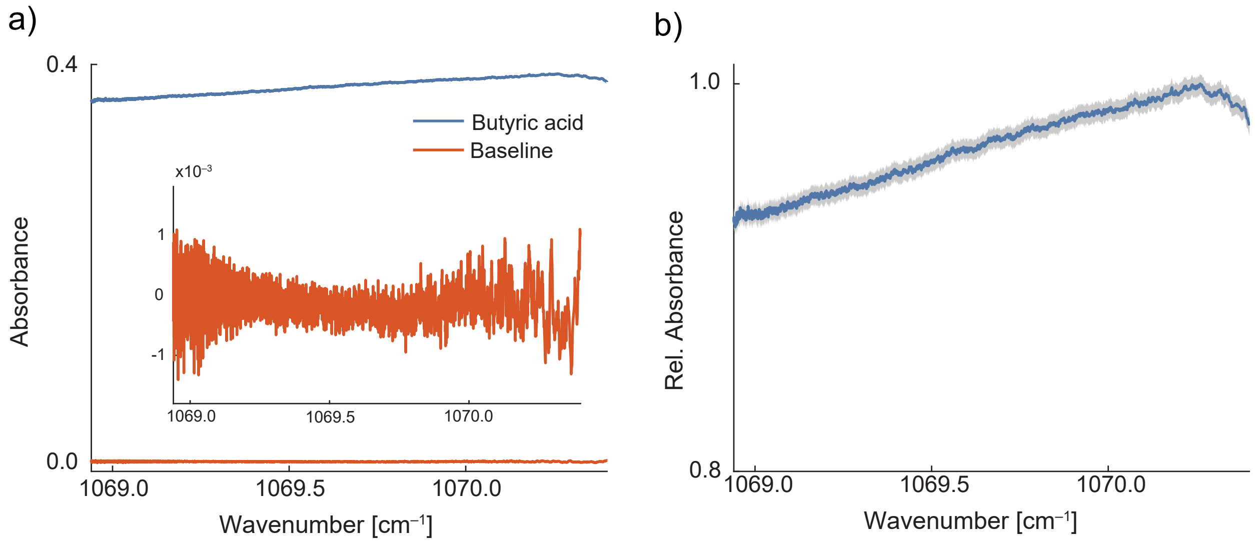

Figure S1: a) Measured absorbance spectrum of butyric acid (blue) and empty-cell-normalized baseline (orange) given on absolute scale. The inset is the zoom-in of the noise floor, indicating an absorbance uncertainty better than 10^{-3}. b) Relative absorbance obtained by diving the measured absorbance with its maximal value (e.g. 0.4 in the case of butyric acid). The gray area represents the 95% confidence interval of the relative absorbance.
